# Potential Role of MSC/Cancer Cell Fusion and EMT for Breast Cancer Stem Cell Formation

**DOI:** 10.3390/cancers11101432

**Published:** 2019-09-25

**Authors:** Ralf Hass, Juliane von der Ohe, Hendrik Ungefroren

**Affiliations:** 1 Biochemistry and Tumor Biology Lab, Department of Obstetrics and Gynecology, Hannover Medical School, 30625 Hannover, Germany; Ohe.Juliane.von.der@mh-hannover.de; 2 First Department of Medicine, University Hospital Schleswig-Holstein, Campus Lübeck, 23538 Lübeck, Germany; Hendrik.Ungefroren@uksh.de; 3 Department of General Surgery, Visceral, Thoracic, Transplantation and Pediatric Surgery, University Hospital Schleswig-Holstein, Campus Kiel, 24105 Kiel, Germany

**Keywords:** epithelial-mesenchymal transition, mesenchymal stem cells, retrodifferentiation, cancer cell fusion, cancer stem cells, tumor therapy

## Abstract

Solid tumors comprise of maturated cancer cells and self-renewing cancer stem-like cells (CSCs), which are associated with various other nontumorigenic cell populations in the tumor microenvironment. In addition to immune cells, endothelial cells, fibroblasts, and further cell types, mesenchymal stroma/stem-like cells (MSC) represent an important cell population recruited to tumor sites and predominantly interacting with the different cancer cells. Breast cancer models were among the first to reveal distinct properties of CSCs, however, the cellular process(es) through which these cells are generated, maintained, and expanded within neoplastic tissues remains incompletely understood. Here, we discuss several possible scenarios that are not mutually exclusive but may even act synergistically: fusion of cancer cells with MSC to yield hybrid cells and/or the induction of epithelial-mesenchymal transition (EMT) in breast cancer cells by MSC, which can relay signals for retrodifferentiation and eventually, the generation of breast CSCs (BCSCs). In either case, the consequences may be promotion of self-renewal capacity, tumor cell plasticity and heterogeneity, an increase in the cancer cells’ invasive and metastatic potential, and the acquisition of resistance mechanisms towards chemo- or radiotherapy. While specific signaling mechanisms involved in each of these properties remain to be elucidated, the present review article focusses on a potential involvement of cancer cell fusion and EMT in the development of breast cancer stem cells.

## 1. Introduction

Tumor tissue and its microenvironment harbors a variety of different cell types which are interacting, proliferating, and differentiating. Among these populations are cancer cells at different stages of malignant development, including a potential subset of proliferation-active cells and cancer cells with self-renewal capacity and stem-like features. Moreover, various immune cell populations, such as CD4^+^ T_H_1 and T_H_2 cells, CD8^+^ cytotoxic T cells, T_reg_ cells, B lymphocytes, dendritic cells, natural killer cells, and monocytes/macrophages, are attracted to the tumor site due to the proinflammatory environment of invasive cancer cell growth [1,2]. An important cell type in the tumor microenvironment is mesenchymal stroma/stem-like cells (MSC), also termed multipotent mesenchymal stromal cells or medicinal signaling cells [3,4]. These cells relay transcellular signaling among the different tumor-associated cell populations by the production and release of trophic factors. In addition, MSC can differentiate into various tumor-associated phenotypes. In general, MSC represent a heterogenous stroma/stem-like phenotype with the capacity to differentiate along the mesodermal lineage by expression of common surface markers, such as CD73, CD90, and CD105 [5,6,7,8]. While MSC primarily reside in perivascular niches of nearly all kinds of human tissues [9,10,11,12,13], these cells differ from pericytes which appear in the vascular walls in the vicinity of endothelial cells and also express some markers in common with MSC [14]. The tissue-specific origins of MSC provide distinct functional characteristics and differences. For instance, bone marrow-derived (BM-)MSC are involved in the regulation of hematopoietic stem cell homeostasis and expansion by providing a hematopoietic stem cell niche [15]. Other physiological functions of MSC include the support of tissue repair [16] and neovascularization [17] at sites of tissue damage. The supportive repair functions of MSC also affect pathophysiological conditions with invasive tumor growth and metastasis and further involve immune-suppressive and anti-inflammatory effects. Mechanistically, MSC in the tumor tissue can interact with immune cells via surface adhesion molecules or cytokines and lectins to down-modulate T cell and natural killer cell activity. Moreover, MSC-mediated release of the macrophage inflammatory proteins MIP-1α/CCL3 and MIP-2α/CXCL2 as well as prostaglandin E2 (PGE_2_) and kynurenine contribute to the conversion of inflammatory M1 macrophages to alternatively activated, immunosuppressive M2 macrophages [18]. In addition, MSC can interact with cancer cells and differentiate into cancer-associated fibroblasts (CAFs) and myofibroblasts, which are important to stabilize tumor tissue at primary and metastatic sites and contribute to chemoresistance [19,20]. MSC also support neovascularization of the tumor tissue [21] via the release of transforming growth factor beta (TGFβ). This growth factor is known to promote invasion and metastasis through the induction of epithelial-mesenchymal transition (EMT), a morphogenetic program during which the epithelial phenotype of the carcinoma cells is lost and replaced with a mesenchymal one [22]. Other reports demonstrated the capacity of MSC to acquire an endothelial phenotype, which is mediated by TGF-β/JNK signaling and negatively regulated by p38α [23,24]. Altogether, enhanced recruitment of MSC and localization in a tumorigenic microenvironment contribute to immune modulation [25], tumor angiogenesis, and various modes of direct and indirect interactions of MSC with tumor cells followed by mutual alterations of their functionalities [26,27,28,29,30].

## 2. Interaction and Cell Fusion of MSC with Breast Cancer Cells

In the course of cellular communication within the tumor tissue, MSC and cancer cells exhibit direct and indirect interactions which involve components of the extracellular matrix and mutual exchange of biologically active compounds and small organelles. In addition to growth factors, cytokines, chemokines, and metabolic products such as PGE_2_, MSC and cancer cells release a variety of extracellular vesicles including microvesicles and exosomes. While the majority of microvesicles are secreted by MSC, these organelles represent useful therapeutic vehicles [31] and can contain distinct proteins as well as RNAs and microRNAs (miRNAs), which contribute to functional changes in both MSC and cancer cells [32]. Alterations in functionality can convert MSC into aberrant cancer-associated and -supporting MSC (CA^+^-MSC) with production of pro-inflammatory cytokines. Conversely, cancer-associated and -suppressing MSC (CA^−^-MSC) are characterized among others by enhanced release of IFNβand/or DKK-1. Therefore, a mixed population of these antagonistically acting aberrant MSC subtypes may either inhibit or support tumor growth [33].

Other cell types, such as tumor-associated macrophages (TAMs) or cancer-associated fibroblasts types besides MSC, can also release trophic factors and interact within the tumor microenvironment, whereby the functionality of cancer cells may be switched eventually resulting in CSC formation and increased tumor heterogeneity [34]. Previous work has demonstrated that the accumulation of pro-inflammatory cytokines, including tumor necrosis factor alpha (TNFα), within the tumor microenvironment enhanced migration, and invasiveness of breast and ovarian cancer cells [35]. Of interest, TNFα can relay signals for direct interactions between MSC and certain breast cancer populations favoring a rare process of MSC/breast cancer cell fusion to result in the formation of breast cancer hybrid cells [36]. Indeed, recent studies substantiated the involvement of TNFα–mediated downstream NFkB activation during MSC/breast cancer cell fusion [37].

While cell fusion can occur as a physiological process, e.g., during formation of myocytes, osteoclasts, or syncytiotrophoblasts among others [38], pathophysiological heterofusion like that of MSC/breast cancer cells is observed within a few minutes and represents a rare event, although the frequency may be higher due to “hidden” fusions [39,40,41,42], and the resulting hybrid cancer cells may gain importance by regulatory advantage following a post-fusion selection process [28]. Hybrid cancer cells have been identified in a variety of different tumor cell types, including breast, ovarian, gastric, and lung cancer, following interaction and subsequent fusion with MSC [26,40,41,43,44].

These hybrid populations increase tumor plasticity, whereby cancer cell fusion can also contribute to enhanced metastases [28,45,46]. The importance of fusion processes for the generation of new cancer cell populations is underscored by the findings that MSC/breast cancer cell fusion can generate various different hybrid subtypes (e.g., MDA-hyb1 to MDA-hyb4). These cells display different tumorigenic potential, altered metastatic behavior, and changes in their sensitivity to chemotherapeutic drugs which elevates tumor heterogeneity and the potential of breast cancer stem cell expansion. However, prolonged growth of hybrid cancer populations is accompanied by post-fusion selection processes to overcome chromosomal instability and to adapt to potential aneuploidy generated by a combined genome of the associated nuclei. Consequently, several hybrid cancer subtypes eventually develop whereby the majority may be eliminated by apoptosis/necroptosis due to regulatory incompatibilities during chromosomal re-arrangement and interference with re-establishing a newly organized cellular functionality. Although developmental pathways and molecular signal transducers for these post-fusion selection processes remain unclear to date, a stabilized hybrid cancer population could evolve from this clearance. Alternatively, a mixture of different hybrid cancer phenotypes arises by carrying altered genomic properties but interdependent growth and survival properties during the clonal convergence of post-fusion selection. Indeed, a comprehensive comparison of karyotypes in various different tumor entities with accompanying subtypes revealed a wide range of aneuploid chromosomes rather than a fixed number of chromosomes [34], substantiating the hypothesis of variable phenotypes by potential mixtures of distinct hybrid cancer cell populations displaying mutually dependent and adaptive expansion properties. Further support is attributed to this hypothesis by the appearance and plasticity of residual cancer cells which remain unresponsive during a tumor-therapeutic approach.

Further mechanisms, such as nanotube formation or trogocytosis, between MSC and cancer cells, can also contribute to mutual genomic alterations [34]. Moreover, cannibalism or entosis represent additional mechanisms for potential hybrid cancer cell formation [34,47]. More importantly, recent observations have documented that MSC/breast cancer cell fusion can also occur in vivo whereby associated processes, including apoptosis and reorganization of the actin cytoskeleton, contribute to an elevated tumor plasticity and heterogeneity with a likely generation of breast cancer stem cells (BCSCs) [48,49,50].

## 3. EMT and the Formation of Breast Cancer Stem Cells (BCSCs) 

In order to fulfill their specialized functions in tissue homeostasis, epithelial cells must be highly differentiated. Maintaining a stable epithelial phenotype requires a series of external cues and may eventually be compromised through the EMT. EMT comes in three different types, termed type-1, -2, and -3: Type-1 EMT occurs during embryogenesis and controls mesodermal and neural tube formation, while type-2 EMT is associated with tissue regeneration/healing and fibrosis. Type-3 EMT occurs in cancer cells following genetic alterations in oncogenes and/or tumor suppressor genes. Carcinoma cells that have undergone EMT are capable of invading surrounding tissues and in combination with the reverse process, mesenchymal-epithelial transition (MET), may contribute to the formation of metastases at remote sites in the body [51,52].

**Figure 1:**
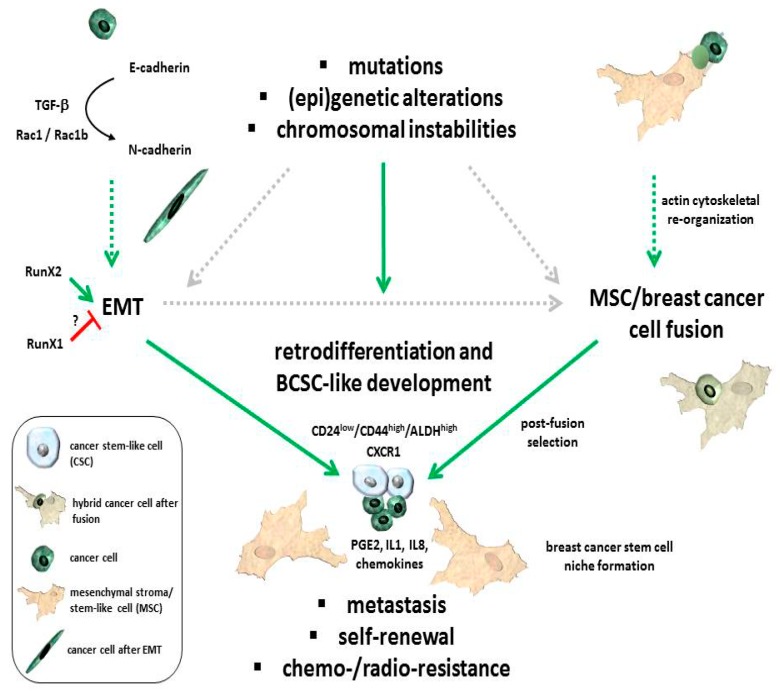
Schematic diagram for a potential development of breast cancer stem cells (BCSCs) via: (1) changes in the DNA structure (mutations, (epi)genetic alterations, chromosomal instabilities); (2) changes in cell fate by epithelial-mesenchymal transition (EMT) including a transforming growth factor beta (TGFβ)-mediated switch of E-cadherin to N-cadherin expression and subsequent induction of EMT-related factors (e.g., Snail, Twist, Vimentin); (3) generation of new cancer cell populations by cell fusion (formation of a fusion-permissive environment by cytoskeletal re-arrangement and distinct physico-chemical parameters (low pH, ionic strength, hydrophilic and lipophilic fluidity etc.) and appropriate arrangement of (glyco)proteins and (glycol)lipids; (4) maintenance of BCSCs in a dynamic breast cancer stem cell niche requiring prostaglandin E2 (PGE_2)_, IL1, IL8, and chemokines among others [101].

During the EMT, epithelial cells lose their contact with the basement membrane and with neighboring cells in the epithelial cell layer by removing tight junction proteins, i.e., E-cadherin, from their cell surface (Figure 1), resulting in the loss of apical−basal polarity. Simultaneously, these cells acquire mesenchymal traits, such as the expression of N-cadherin and Vimentin (Figure 1), a spindle-shaped morphology, increased motility, and resistance to induction of apoptotic cell death. EMT can be triggered by external conditions like physical stress (e.g., ionizing radiation), hypoxia, nutrient deprivation, hyperglycemia, or tumor−stromal cell interactions, including biochemical stimuli such as growth factors. In the course of EMT, specific transcription factors, such as snail family transcriptional repressor 1 (Snail (Snai1)), snail family transcriptional repressor 2 (Snai2 (Slug), Twist-related protein 1 (Twist1), Zinc finger E-Box binding homeobox (Zeb)1, and/or Zeb2, are induced in response to certain ligands, including TGFβ, Notch (neurogenic locus notch homolog protein), and members of the Wnt family of proteins [53,54,55]. The transcription factors downregulate the expression of *CDH1* (the gene for E-cadherin) and other genes encoding epithelial proteins and upregulate the expression of mesenchymal marker genes. The loss of E-cadherin, which can occur by either transcriptional silencing or protein internalization (see below), is a hallmark of EMT [51].

It has been observed that EMT may proceed to a partial or complete mesenchymal phenotype. Thus, cells may retain some epithelial characteristics resulting in mixed or intermediate phenotypes, a phenomenon referred to as “partial EMT” [56]. Many in vitro studies have demonstrated that the EMT process is regulated at the transcriptional level, i.e., through silencing of *CDH1*. By employing a lineage-labeled *LSL-Kras^G12D/+^*; *Trp53^fl/+^*; *Pdx1-Cre* (KPC) mouse model (LSL-Kras^G12D^; P53^loxP/+^; Pdx1-cre; LSL-Rosa26^YFP/YFP^) of pancreatic ductal adenocarcinoma (PDAC) to study EMT in vivo, Aiello et al. [57] found that loss of the epithelial phenotype in many tumors was accomplished through protein internalization, resulting in a partial EMT. In contrast, cells that primarily use transcriptional repression of *CDH11* and other epithelial genes experience a complete EMT. Intriguingly, carcinoma cells which have undergone a partial EMT migrate as clusters (also termed collective-cell migration), as opposed to the single-cell mode of migration which is associated with a complete EMT. This alternative program to undergo EMT is not restricted to cells of pancreatic origin but is also seen in many breast cancer cell lines. This suggests that carcinoma cells have different routes of losing their epithelial phenotype, which in turn determines their mode of invasion and dissemination [57]. Partial EMT is also a physiological process that occurs during branching morphogenesis of the mammary gland. Here, the progenitor cells lose their polarity and transiently acquire a mesenchymal phenotype [58] associated with upregulation of Snail (Snai1 zinc finger transcriptional repressor) and Twist (basic helix-loop-helix transcriptional factor) expression [59]. Maintaining some epithelial characteristics by inhibiting EMT at terminal end buds, i.e., through activation of the transcription factors Elf5 [60] and Ovol2 [61], is a crucial event during mammary gland development.

As mentioned above, cancer cells exploit the EMT process to become invasive and eventually metastatic. In breast cancer, support for this comes from the finding that EMT increases during the progression of ductal carcinoma in situ to invasive basal-like breast cancer [62]. Moreover, characteristics of EMT are more prevalent in the “basal-like” and “claudin-low” breast cancer histological subtypes than in the “luminal A/B” subtypes [63]. Given the proposed causative role of tumor cell EMT for invasion and metastasis, this may provide an explanation for why basal and claudin-low subtypes are more metastatic. The knockout or knockdown of Snail, Twist, or Zeb1/2 in human or murine cancer cells resulted in strong inhibition of their metastatic potential in vivo [53,64,65]. For instance, depleting Snail in MMTV-PyMT mice prevented nearly all metastatic spread to the lung, while activating EMT in human breast cancer cells enhanced metastasis [64].

Earlier studies focused on the genes that suppress epithelial gene expression and promote activation of EMT and the mesenchymal phenotype [53], i.e., RUNX2 (Runt-related transcription factor 2) [66]. More recent studies have revealed a couple of proteins that act to sustain the epithelial phenotype and thereby prevent EMT [22]. For the establishment and maintenance of the epithelial phenotype in mammary epithelial cells, and the suppression of tumor growth, RUNX1 appears to be required as concluded from the consequences of a RUNX1 knockdown in mammary cells [22,67]. Our own studies in human PDAC have shown that RAC1B, a splice variant of the small GTPase RAC1, may yet represent another member of these “guardians” of the epithelial phenotype based on the observations that RAC1B promoted the expression of E-cadherin and protected pancreatic cancer cells from TGFβ1-induced EMT [68].

While EMT-associated changes have been well documented in vitro and even in some mouse models of cancer, the significance of EMT programs driving EMT in physiological contexts, i.e., during tumor progression and metastatic dissemination, still remains a matter of debate. Two reports claimed that EMT was not required for invasion and metastasis [69,70]. In one of these studies, a murine breast cancer model with spontaneous metastasis to the lung was employed to show that many cells, despite being negative for Fsp1 (fibroblast-specific protein 1), managed to metastasize to the lung, leading the authors to conclude that EMT was not necessary for metastasis to occur [69]. In another study, Zheng et al. knocked down either Snail or Twist in the KPC model of spontaneous PDAC (see above) but failed to observe any differences in metastasis when using α-SMA (α-smooth muscle actin) as an EMT marker for tracing the tumor cells [70]. The seemingly contradictory results may, apart from the different cancer types analyzed, be explained by the use of different (and not firmly established) EMT markers to trace the metastatic cells [57]. Furthermore, two other reports which employed the same KPC mouse model, as well as the majority of other studies, are in favor of a crucial role for EMT in driving the metastatic process in vivo. Krebs et al. were able to show that depletion of Zeb1 suppressed stemness, invasion, and metastatic spread [71], while Aiello et al. demonstrated that partial and complete EMT were strictly associated with collective and single-cell migration modes, respectively [57].

Of note, the two studies that denied a role of EMT in metastasis showed that EMT is nevertheless required in order for the tumor cells to become resistant to chemotherapeutic drugs [69,70]. A series of other studies also demonstrated that induction of EMT conferred resistance to endocrine therapy (letrozole) [72], radiotherapy [73,74], and chemotherapy (docetaxel, trastuzumab) [75]. To date, the molecular mechanisms underlying this phenomenon remain enigmatic but may include induction of quiescence, at least in EMT induced by TGFβ [76].

Development of BCSCs by MSC-mediated enhanced release of chemokines including CCL5/RANTES and subsequent activation of cancer cell dissemination and metastasis can also be triggered via MSC-stimulated expression of the EMT markers Snail, Twist, Vimentin, and N-cadherin in breast cancer cells [39]. The EMT process has also been strongly implicated in the formation of BCSCs [52,77]. In their pioneering study, Mani et al. provided convincing experimental evidence for a direct link between the EMT and the gain of stem cell features by showing that EMT induction after long-term treatment with TGFβ in human mammary epithelial cells (HMEC) resulted in the expression of stem cell markers as well as an increased sphere-forming ability. Conversely, stem cell-like cells derived from cultured HMEC, murine or human mammary glands, or breast cancer tissue expressed markers of EMT, while transformed HMEC that underwent an EMT had an increased sphere and colony-forming ability in vitro, and tumor-generating ability in vivo [78]. Mechanistically, the link between EMT and CSCs is controlled by a regulatory network involving EMT-associated transcription factors and epigenetic regulators. For instance, in mouse and human breast cancer cells, Twist interacts with several components of the Mi2/nucleosome remodeling and deacetylase (Mi2/NuRD) complex for subsequent recruitment to the *CDH1* promoter and transcriptional repression [79]. In addition, the functional association between EMT and CSC generation is governed by the finely tuned balance of complex reciprocal feedback loops between Zeb family members (Zeb1/2) and the miR-200 family of miRNAs. In breast cancer, miR-200 suppresses the ability of normal mammary stem cells to form mammary ducts, and inhibits tumor formation driven by human BCSCs in vivo [80]. MiR-200 normally promotes epithelial differentiation and blocks the translation of stem cell-associated factors and epigenetic regulators such as BMI1 (B lymphoma Mo-MLV insertion region 1 homolog) by targeting its own repressor, Zeb1 [80]. Conversely, Zeb1 can induce EMT and a CSC-like phenotype by directly inhibiting the expression of its own repressor, miR-200 [80]. Another reciprocal feedback loop involves miR-34, Snail, and p53. MiR-34, which is under positive control by p53, can inhibit upregulation of Snail, and induction of EMT, migration, and invasion, whereas Snail can suppress the transcription of miR-34 family members [81]. Moreover, Snail can suppress p53 by promoting its deacetylation and subsequent proteasomal degradation, resulting in the expansion of tumor-initiating cells in human breast cancer [82]. The observation that miR-200 and Snail also display antagonistic effects in EMT induction [83] adds another level of complexity to the system in that the miR-200-Zeb and the miR-34-Snail double-negative feedback loops are connected via miR-200 to regulate EMT, cell motility, and stemness [84].

The tumor microenvironment is crucially involved in EMT and CSC generation, maintenance, and expansion. In a pro-inflammatory environment, i.e., under conditions of chronic inflammation, inflammatory mediators such as cytokines and chemokines (TGFβ, TNFα, IL1, IL6, IL8) produced by immune cells and MSC also induce tumor cell EMT and impact CSCs [85,86]. TNFα, IL1, and IL6 operate mainly through activation of canonical NFκB signaling. Since NFκB activation can upregulate Snail [87], these cytokines may directly promote the Snail-dependent effects described above, i.e., suppression of p53 and miR-34, resulting in EMT, cell motility, CSC formation, and metastasis [81]. In addition to transcription factors and NFκB, EMT and CSC biology are controlled by various other signaling pathways, such as Notch, Hedgehog, Wnt/β-catenin, and TGFβ/Smad. 

Above, we discussed available work demonstrating a crucial role of EMT in metastasis and chemoresistance. Intriguingly, a couple of studies have suggested that BCSCs that are generated in the course of the EMT process are the cellular mediators that not only account for metastatic outgrowth, but also chemoresistance, as they are unresponsive to radiation and chemotherapy, and contribute to relapse [88,89,90].

The phenotypic characterization of BCSCs revealed that these cells express high levels and activity of the cell-surface glycoprotein CD44 (the hyaluronan receptor) and ALDH-1 (aldehyde dehydrogenase-1), respectively, as well as low levels of another cell-surface glycoprotein, the GPI-anchored sialoglycoprotein CD24 (CD24^low^/CD44^high^/ALDH^high^) (Figure 1). Cells displaying this surface antigen expression profile may act as tumor-initiating cells [91] and contribute to intratumor heterogeneity. Moreover, results from clinical studies have shown that the (CD24^low^/CD44^high^/ALDH^high^) phenotype was preferentially associated with triple negative breast cancer (ER/PR^/^HER2^−^) and an increased risk for metastatic dissemination compared with other breast cancer types [92,93]. BCSCs have been demonstrated to be phenotypically heterogeneous, possessing either a mesenchymal- or an epithelial-like phenotype. Mesenchymal-like BCSCs are primarily quiescent and are located at the invasive front of the tumor, while their epithelial-like counterparts are characterized by expression of ALDH-1, active proliferation, and are localized more centrally within the tumor tissue. Interestingly, the gene signatures of mesenchymal-like and epithelial-like BCSCs are quite similar when compared among the various molecular subtypes of breast cancer. Therefore, BCSCs may exhibit a certain extent of plasticity which permits transition between a mesenchymal (EMT) state, to enable these cells to invade adjacent tissues and disseminate to distant sites, and an epithelial (MET) state, which later in the metastatic cascade allows for tumor outgrowth at metastatic sites [94]. This process is regulated by the tumor microenvironment. As certain MSC subpopulations contribute to induction of EMT in breast cancer cells [39], it remains to be determined if switches between these two states of BCSCs can also be regulated by MSC. Since many breast cancer cell lines also utilize the alternative EMT program discussed above [57], an interesting issue relates to the question as to whether BCSCs can be generated during both partial and complete EMT and, if so, whether they differ in phenotype and/or function.

## 4. Breast Cancer Stem Cell Properties and Corresponding Carcinoma Stem Cell Niche 

Transformation of normal stem or progenitor cells in the mammary gland by the deregulation of self-renewal pathways including Hedgehog, Notch, and Wnt/β-catenin signaling via the transcription factor Bmi-1 can promote the appearance of tumorigenic BCSCs [95]. In addition, development of cancer stem-like cells including those from liver and breast tissue may involve retrodifferentiation, fusion or EMT of maturated tumor cells or tumor-associated cells by acquisition of self-renewal capacity, and exhibiting anti-apoptotic mechanisms for maintenance of a neoplastic environment [34,96]. The process of retrodifferentiation describes a reversion of all differentiated markers by the development of a stem-like phenotype which includes retrograde senescence [97]. As a consequence, retrodifferentiated and rejuvenated stem-like cancer cells display the potential of self-renewal (Figure 1). Likewise, BCSCs of the CD24^low^/CD44^high^ phenotype display tumorigenic potential and self-renewal capacity [91,98] and may evolve by retrodifferentiation. Moreover, increased ALDH-1 activity was identified in HMEC associated with stem/progenitor properties whereby tumorigenic breast cell subsets with increased ALDH-1 expression and activity exhibit self-renewal capacity and initiation of tumor growth [99] (Figure 1).

**Figure 2:**
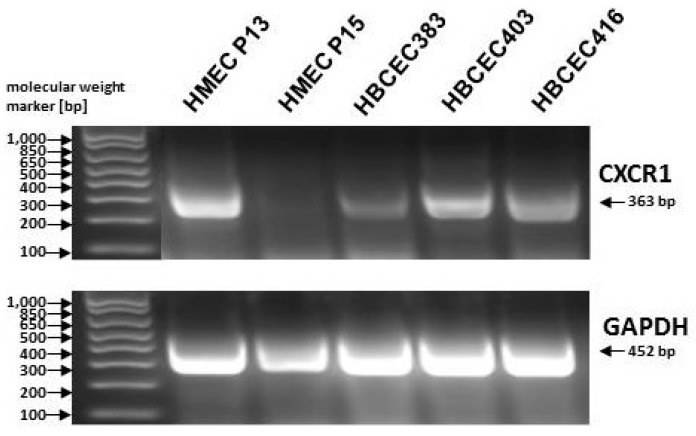
Detection of the IL-8 receptor, CXCR1, by RT-PCR in young proliferating human mammary epithelial cells (HMEC) in passage 13 and in three different patient-derived human breast cancer epithelial cell (HBCEC) populations in contrast to growth-arrested senescent HMEC in passage 15 [104]. The expression of CXCR1 is a phenotypic feature of BCSCs since these cells were reported to depend on IL8 signaling for survival [102]. Amplification of glyceraldehyde-3-phosphate-dehydrogenase (GAPDH) transcripts (amplification product: 452 bp, [28]) served as a control. The PCR primers used were: CXCR1 sense: 5′-GGG GCC ACA CCA ACC TTC-3′, antisense: 5′-AGT GCC TGC CTC AAT GTC TCC-3′, amplification product: 363 base pairs (bp). Molecular weight is represented by the DNA ladder standard (Invitrogen/ThermoFisher Scientific, Carlsbad, CA, USA).

The development of cancer cell stemness can occur by different mechanisms, such as fusion or EMT which involve (epi)genetic alterations and chromosomal re-arrangements, eventually triggering a retrodifferentiation program. The subsequent release of trophic factors and a variety of cytokines and chemokines by MSC contribute to enhanced proliferation of breast cancer cells, including an elevated amount of BCSCs. Maintenance and progressive expansion of BCSCs may require the establishment of a cancer stem cell niche (CSCN) with a timely available concentration of trophic factors predominantly provided by appropriately primed MSC populations [100] (Figure 1). Certain metabolic and regulatory requirements have been suggested which favor the establishment of a CSCN. Therefore, PGE_2_ production by MSC, paralleled by cancer cell- and immune cell-derived IL1, further stimulates MSC to secrete a panel of cytokines, which, in turn, together with PGE_2_, builds a local gradient to confer Wnt/Frizzled/β-catenin signaling in the cancer cells, thereby contributing to the development of CSCs [101]. Moreover, IL8 signaling via the receptor CXCR1 appears crucial for the maintenance of certain BCSC subtypes since inhibition of the receptor downstream signaling by interfering agents for ligand binding was associated with massive apoptosis/necroptosis and a subsequent decrease in the breast cancer stem-like cell population [102] (Figure 1, Figure 2). The activation of IL8 signaling in BCSCs is substantiated by visualizing the formation of a putative breast CSCN. Long-term cultivation of primary human breast cancer epithelial cells (HBCEC) derived from breast cancer patient’s biopsies [103] is developing small proliferative active cells as putative BCSCs which are circularly surrounded by larger growth-reduced and senescent cells demonstrating an exchange of small vesicles [100]. Moreover, marked IL8 expression was detectable in these human in vitro breast CSCN in contrast to nontumorigenic normal HMEC, suggesting that interfering with CXCR1 expression and/or activation represents a potential therapeutic approach in targeting BCSCs (Figure 2).

Of interest, these mechanisms of DNA re-structure and cellular changes can also lead to apoptosis/necroptosis. Thus, genetic alterations and chromosomal instabilities can induce DNA repair processes via ATM/ATR and downstream signaling eventually resulting in p53- or DNA damage-mediated cell death. Moreover, cell fusion requires chromosomal and functional nuclear re-organization whereby the majority of the generated new cancer hybrid cells are dying during a post-fusion selection process due to regulatory incompatibility of the two nuclei.

## 5. Conclusions

Although it is commonly accepted that BCSCs are crucially involved in driving tumor progression and resistance to therapy, mechanistic insights into how these cells are generated and maintained in the cancer tissue are comparatively scarce. Here, we discussed a contribution of MSC to enhanced breast cancer proliferation which may also promote BCSC generation by several mechanisms including fusion with cancer cells, retrodifferentiation of differentiated cancer cells or hybrids, or, indirectly, via EMT induction. Moreover, maintenance and expansion of BCSCs may require the establishment of a cancer stem cell niche with potential involvement of MSC.

Although CSC generation is thought to be closely associated with EMT on the one hand and self-renewal, metastasis, and therapy resistance on the other hand, it is still a matter of debate whether EMT is a prerequisite for metastasis to occur. Elucidating the precise role of EMT or cell fusion in CSC formation, and how MSC impact both processes, may provide novel opportunities to target BCSCs in breast cancer therapy. Certain therapeutic strategies against BCSCs are a matter of debate [31,105,106,107,108,109].

## Abbreviations

ALDH-1	aldehyde dehydrogenase-1
α-SMA	α-smooth muscle actin
(B)CSCs	(breast) cancer stem cells
CA^+^-MSC	cancer-associated and -supporting MSC
CA^-^-MSC	cancer-associated and -suppressing MSC
CCL5	CC-chemokine ligand 5
CSCN	cancer stem cell niche
CXCR1	CXC-motif chemokine receptor 1
DKK-1	Dickkopf-related protein 1
EMT	epithelial-mesenchymal transition
EGF	epidermal growth factor
Fsp 1	fibroblast-specific protein 1
GAPDH	glyceraldehyde-3-phosphate-dehydrogenase
HBCEC	human breast cancer-derived epithelial cells
HGF	hepatocyte growth factor
HMEC	human mammary epithelial cells
IFNβ	interferon β
IL1	interleukin 1
IL8	interleukin 8
MSC	mesenchymal stroma/stem-like cells
MET	mesenchymal-epithelial transition
microRNA	miRNA
PDAC	pancreatic ductal adenocarcinoma
PGE_2_	prostaglandin E2
RUNX1, 2	runt-related transcription factor 1, 2
TAMs	tumor-associated macrophages
TGFβ	transforming growth factor-beta
TNFα	tumor necrosis factor alpha
Zeb	Zinc-finger E-box homeobox
